# *In Vitro* and* In Vivo* Anti-Salmonella Evaluation of Pectin Extracts and Hydrolysates from “Cas Mango” (*Spondias dulcis*)

**DOI:** 10.1155/2019/3578402

**Published:** 2019-04-23

**Authors:** Denis Zofou, Golda Lum Shu, Josepha Foba-Tendo, Merveille Octavie Tabouguia, Jules-Clement N. Assob

**Affiliations:** ^1^Medical Research and Applied Biochemistry Laboratory (Drug Discovery and Development Research Unit), University of Buea, Cameroon; ^2^Department of Biochemistry and Molecular Biology, Faculty of Science, University of Buea, Cameroon; ^3^Department of Chemistry, Faculty of Science, University of Buea, Cameroon; ^4^Department of Biochemistry, Faculty of Science, University of Yaounde 1, Cameroon; ^5^Faculty of Health Sciences, University of Buea, Cameroon

## Abstract

**Background:**

The threat to human health posed by multidrug-resistant strains of S*almonella typhi *(*S. typhi*) and S*almonella paratyphi *(*S. paratyphi*) is of growing concern. Generally, there has been increasing resistance and even multidrug resistance to almost all classes of antibiotics. This has rendered treatment with antibiotics difficult and costly. The present study investigated the bioactivity of pectin and pectin hydrolysates derived from a local fruit,* Spondias dulcis*, against four strains of* Salmonellae*.

**Methods:**

Pectin was extracted from alcohol extractives-free peel by acidic hydrolysis at a temperature of 80°C for one hour at pH 2 and 4. The pectin was precipitated with 95% alcohol at an extract to alcohol ratio of 1:10 v/v. Antimicrobial activity was determined using agar well diffusion technique. The minimum inhibitory concentration (MIC) and minimum bactericidal concentration (MBC) values were determined using the broth dilution technique. An* in vivo* study was then carried out with the bioactive extracts against the most resistant bacteria strain, to fully establish the therapeutic effect of these extracts. Balb/C mice were used, and ciprofloxacin was the positive control antibiotic. The extracts were administered to mice at two doses, 5mg/Kg and 10mg/Kg. The efficacy of extracts in the treatment of typhoid was evaluated based on survival rate, change in body weight, and change in bacteria load.

**Results:**

Only one of the extracts (crude pectin pH 2.5) was active against all the* Salmonellae *by well diffusion, and the growth inhibition varied from 12mm to 15mm at100 *μ*g/ml. Three of the extracts (crude pectin pH 2.5, pH 4, 12h hydrolysate, and pH 4, 1h hydrolysate) had MIC and MBC against all four* Salmonellae* strains with MIC ranging from 5.68 to 44.45 *μ*g/ml and MBC from 11.36 to 44.45 *μ*g/mL. Three treatments, namely, the pH4-12 hr, hydrolysate at 10mg/Kg and 5mg/Kg, and the pH4-1hr, hydrolysate at 10mg/Kg, had therapeutic effects against Salmonella infection in mice.

**Conclusion:**

The present study highlights the potential of pectin oligosaccharides as new source of anti-Salmonella drugs. Further investigations including exploration of mechanism of action of the most active pectin extracts/hydrolysates are envisaged.

## 1. Introduction

Typhoid fever is a systemic prolonged febrile illness caused by certain* Salmonella* serotypes.* S*erovars* Typhi *and* Paratyphi *of this serotype,* Salmonella enteric *subspecies* enterica, *are the etiologic agents that cause typhoid and paratyphoid fevers, respectively. Typhoid fever is encountered worldwide but primarily prevalent in developing countries. The risk factors for typhoid are similar to those of cholera and other epidemic prone diarrheal diseases and are mainly related to access to safe water and the functionality of sanitation systems as well as food safety. It commonly presents with a sudden onset of fever, headache, abdominal pain, and diarrhea and can quickly progress to a variety of potentially fatal complications, including gastrointestinal haemorrhage and intestinal perforation. Even though the infection is often curable with antibiotics, the growing prevalence of resistance makes vaccination of vulnerable groups an increasingly urgent priority and the search for new antimicrobial agents against* Salmonella *species.

Pectin is a complex mixture of polysaccharides that makes up about one-third of the cell wall dry substances of higher plants [1]. Although pectin was discovered over 220 years ago, its structure and composition are not yet completely elucidated [2]. This is probably because pectin structure can change during extraction from plants, storage, and processing. Presently, pectin is thought to be mainly composed of 1-4 linked *α*-D-galacturonic acid [3]. Plant fruits, both pulp and the rind, generally contain the highest amounts of pectin compared to other parts of plants. Citrus peel and apple pomace are currently the main source of commercial pectin. Pomace has about 15% of wet pectin while citrus peel such as orange and grape fruit has 30% of wet pectin [4]. The use of pectin and pectin oligosaccharides in the prevention and treatment of human ailments is a recent practice. Pectin and combinations of pectin with other colloids have been used extensively to treat diarrheal diseases, especially in infants and children [5]. Pectins are found to have antimicrobial potential against the methicillin resistant* Staphylococcus aureus* and* Aspergillus niger *[6]. Pectin also showed antibacterial activity against Helicobacter pylori [7].

Pectin oligosaccharides are produced from pectin by enzymatic treatment or acid hydrolysis, which for the most part is composed of galacturonic acid, with few rhamnose units in between, from where the arabinan and galactan units branch out [8]. The structure is based on the source and the processing treatment [9]. Functional oligosaccharides have been found effective in gastrointestinal normal flora proliferation and pathogen suppression, dental caries prevention, enhancement of immunity, facilitation of mineral absorption, source of antioxidant, antibiotic alternative, regulators of blood glucose in diabetics, and serum lipids in hyperlipidemics [5]. Xylooligosaccharides in pharmaceutical preparations exhibit antiallergy, antimicrobial, anti-infection, and anti-inflammatory properties, selective cytotoxicity, and immunomodulatory action [10].

*Spondias dulcis* (synonym:* Spondias cytherea) *commonly known as ambarella and “cas mango” in Cameroon is a tropical tree with edible fruits containing a fibrous pit. This fast-growing plant is usually between 10 and 12 meters high.* S. dulcis* has deciduous, pinnate leaves, 20–60 cm in length, composed of 9 to 25 glossy, elliptic, or obovate-oblong leaflets 9–10 cm long, which are finely toothed toward the apex.* S. dulcis* has diverse traditional uses of its fruits, leaves, and bark in different parts of the world. Its leaves and shoots are used as a remedy for diarrhea [11]. There has been an extensive review on the medicinal properties of* S. dulcis*. The roots, leaves, flowers, fruits, and especially bark are used to treat myriad medical problems such as wounds, fever dysentery, vaginal bleeding, genital ulcers, respiratory conditions, and intestinal and digestive ailments [12].* S. dulcis* peel is a major source of pectin, which is generally discarded as being of no nutritional use.

This study was therefore prompted, aiming at exploring pectin extract and oligosaccharides (pectin hydrolysates) obtained from the peel of a local Cameroonian fruit of* Spondias dulcis* as a potential source of anti-*Salmonella *therapy.

## 2. Materials and Methods

All reagents used were obtained from Sigma Aldrich, Germany.

### 2.1. Acquisition of Fruits

The cas mango fruits were obtained from a local market in Douala Cameroon. Fruits were carried in sterile bags and stored in the refrigerator at 4°C prior to its use. The fruits were then washed with tap water and then rewashed with 20% detergent in distilled water and rinsed twice in distilled water.

### 2.2. Extraction of Pectin

The procedure for pectin extraction was designed such that the yield of pectin in two different extraction conditions could be compared [2].

The fruit peels were weighed and blended with water in a ratio of 1:2 (w/v). The sludge was transferred into two 1500 ml beakers and the p^H^ adjusted to 2.5 and 4 using 0.5M HCl and 0.5M NaOH. A volume of 500 mL portions of the sludge was refluxed at 70°C for 30 minutes and allowed to cool. The sludge was filtered using a moistened cheese cloth, ensuring the removal of maximum liquid. The volume of filtrate obtained was noted. Boiling 95% ethanol (about 60-80°C) was then added to the filtrate in a ratio of 1:1(v/v) and left to stand for 15 minutes. Pectin was obtained as a gelatinous precipitate. This precipitate was filtered with Watman filter paper number 1. The same procedure was followed for all other batches of sludge. Pectin precipitates were stored at 4°C.

### 2.3. Purification of Pectin

In order to purify the pectin, it was redissolved with distilled water in a ratio of 1:2 (w/v) at 100°C in a water bath. The dissolved pectin was then reprecipitated with 95% boiling ethanol in a ratio of 1:1(w/v) and filtered with Whatman filter paper number 1. This process was repeated trice and the purified pectin stored at 4°C in air tight containers in order to preserve the quality.

Fifty grams (50 g) of crude pectin and purified pectin was oven dried over at 60°C in order to determine the actual amount of dry pectin which can be derived from the pectin gel.

### 2.4. Identification of Pectin

The extracted pectin was qualitatively determined by the following tests.

### 2.5. Test with 95% Ethanol

On adding an equal volume of ethanol (95%) to 1% w/v solution of pectin sample, a gelatinous precipitate produced was taken as positive test [13].

### 2.6. Test with Potassium Hydroxide (KOH)

To 5ml of a 1% w/v solution of pectin sample, 1ml of a 2% w/v solution of KOH was added and set aside for 15 minutes. A transparent semigel will be produced. When the above gel is acidified with dilute HCl and shaken well, a gelatinous precipitate is formed. Upon boiling, this precipitate will became white and flocculent.

### 2.7. Pectin Hydrolysis

Mixtures of pectin hydrolysates were obtained by incubating “ambarella” pectin in Erlenmeyer flasks in a water (35°C) bath with shaking, with an appropriate amount of endopolygalacturonase (EPG) at 35°C for different reaction times. The reaction mixtures contained 1% pectin solution (w/v), 0.15 M NaCl in acetate buffer (0.05 M), and 0.15 mg ml-1 of endopolygalacturonase (w/v) and the pH adjusted to 6 using HCl and NaOH. In order to obtain different types of pectin hydrolysates, the following conditions were applied: (i) to obtain from pentamers to octamers, the reaction mixture was incubated in the water bath at (35°C) for 1 hour at pH 6.0; (ii) for dimer to pentamer the reaction mixture was incubated in the water bath at (35°C) for 12 hours at pH 6.0. In both cases, the enzymatic reactions were terminated by heating the solutions for 5 minutes at 100°C. Commercial pectin was used as a control. The KOH test for pectin was used to confirm the complete degeneration of pectin by the enzyme.

### 2.8. Antimicrobial Activities

#### 2.8.1. Microorganisms and Culture Media

Four bacterial strains were used for this study, 3 laboratory strains, and one clinical strain. The laboratory strains (*Salmonella enterica typhimurium* ATTC 2680,* Salmonella enterica typhimurium* ATTC 2488, and* Salmonella choleraesuis*) from the American Type Culture Collection (ATCC) were kindly donated by BEI-Resources, Manassas, USA. Nutrient agar was used for activation and routine maintenance of the strains.

The clinical strain of* Salmonella* was obtained from Buea Regional Hospital Laboratory, subcultured several times on* Salmonella Shigella* (SS) agar to obtain pure and single bacterial colonies. The colonies were subcultured on Nutrient agar and characterized using Kliger Iron Agar (KIA).

#### 2.8.2. Determination of* In Vitro *Anti-Salmonella Activity of Pectin and Pectin Hydrolysates

The extracted pectin in solution and the pool of unfractionated hydrolysates were evaluated for* in vitro *antimicrobial activity at 100*μ*g/ml

*(a) Inhibition Zone Diameters*. These tests were performed using the agar well diffusion method described by Tabouguia* et al.* [14], with some modifications. The inocula were prepared by dissolving 3 to 5 bacterial colonies from 24-hour culture of Nutrient agar in sterile saline (0.9% NaCl). The turbidity was then adjusted to match 0.5 McFarl and standard turbidity (10^8^ CFU/ml). Muller Hinton Agar (20ml) was poured into each of the 90mm Petri dishes. The bacterial suspension (100 *μ*l) was uniformly spread on an MH medium using a Pasteur pipette and allowed to dry. Using a sterile borer, 6mm diameter wells were bore on the agar. 60 *μ*l, of each of the test solutions, and the negative control (60 *μ*l of saline) and positive controls (ceftriaxone) were carefully added into designated wells on the surface of the agar containing the bacteria. The plates were kept for 30 minutes and then incubated at 37±1°C for 24h. The plates were examined for growth and the zones of inhibition measured using a ruler. The endpoint of inhibition is where growth starts.

#### 2.8.3. Determination of MIC and MBC

The broth dilution method [14] was adopted for determination of MIC and MBC values against the pathogens, using 96 well plates. The pectin extracts and hydrolysates (100*μ*g/ml) were serially diluted in different aliquots and the final volumes of the aliquots with Muller Hinton Broth. Equal amount of the specific pathogen (50*μ*l) at McFarland's turbidity was added in different aliquots and the well plates were kept for 24h at 37±1°C. The minimum concentration of the pectin extract that kills the bacterial was taken as MBC (Minimum Bacterial Concentration) while the minimum concentration that inhibits the growth of the organism was taken as MIC.

#### 2.8.4. *In Vivo* Anti-Salmonella Activity of Preselected Extracts and Hydrolysates in Mouse Model

From the results of the* in vitro* antibacterial assay, one extract and two hydrolysates with significant activity were considered for* in vivo* testing against* Salmonella enterica typhimurium* ATTC 2860 Balb/c mouse model.

#### 2.8.5. Ethical Considerations

The experiment was performed in accordance with ethical guidelines for the care and use of animals in research. The mice were kept in sterile mice cages at room temperature (27 ± 2°C), and a 12-hour cycle of light and dark. They were given access to animal feed pellet and water* ad libitum*. The proposal, describing the handling and treatment of animals, was reviewed and approved by the University of Buea Ethical Review Board for the Use of Animals in Research (UB-IACUC).

#### 2.8.6. Infection and Treatment of Mice [15]

Forty-five Balb/c strain albino mice, all male, weight between 30 and 40g from the animal breeding unit of the Medical Research and Applied Biochemistry Laboratory, University of Buea, were used in the study. The mice body surface was wiped with 70% alcohol, and the animals were confirmed for the absence of any enteric infection using stool culture in both Mueller Hinton and* Salmonella Shigella *agar. The faeces collected were dissolved in saline distilled water (0.9% NaCl) at a proportion of 0.5g for 1mL of suspension. Aliquots (100 *μ*L) of fecal suspensions in saline distilled water (0.9% NaCl) were then plated on* Salmonella Shigella *agar. The plates were incubated overnight at 37°C. Typical colonies were then identified and counted on the plates. For plates whose colonies were uncountable, the bacterial suspensions were serially diluted and cultured. The CFUs obtained from dilution were then multiplied by the dilution factor. Any animals found infected prior to the study were excluded from the study.

The animals to be infected were fasted for 3 hours (Groups A to G and I) and given by gavage 1mL of saline solution (0.9% NaCl) containing 3.0×10^8^ CFU of the test microorganisms.

The animals were then maintained at room temperature [(23 ± 2)°C] with a 12 h light-dark cycle and standard animal feed and water were provided and feeding habits were monitored. To verify that infection, the bacterial load in the faeces of the animals was determined one day before infection and five and seven days following the infection, by stool culture as described above. Typical colonies were then counted.  Table 1 shows how mice were distributed.

### 2.9. Statistical Analysis

Data obtained was expressed as mean ± SDEV. The different treatments were compared against controls and against each other using the Wilcoxon signed ranks test at 95% confidence intervals. Survival rate was expressed in percentages. Statistical Package for Social Sciences (SPSS) version 21.0 was used.

## 3. Results

*Yield of Pectin extracted and physicochemical characteristics of pectin extracts and their hydrolysates*: the extraction yield was higher (6.98%) at pH 2.5, compared to 4.59 obtained at pH4.  Table 2 summarizes the physicochemical characteristics of the different pectin extracts and their hydrolysates.

The same tests were used to qualitatively confirm hydrolysis and negative results were obtained.

### 3.1. Anti-*Salmonella* Activity of Pectin Extracts

#### 3.1.1. *In Vitro* Activity

Table 3 summarizes the findings on dish diffusion test of the different pectin extracts and hydrolysates. The zones of inhibitions varied from 12 mm to 15.0 mm at 100 *μ*g/ml.* Salmonella *clinical was most inhibited with an inhibition zone of 15 mm while* Salmonella enterica typhimurium* ATTC 2860 recorded the least zone of inhibition, 12mm.

*Diameter of the wells*: 6mm, all extracts which had a diameter zone of inhibition of 6mm were inactive on the bacteria.

### 3.2. Result of the Microplate Dilution Tests

The values of minimum inhibitory concentration (MIC) of the different extracts and hydrolysates are shown in Table 4.

### 3.3. *In Vivo* Anti-Salmonella Activity of Pectin and Hydrolysates in Mice

The three extracts which had significant MICs (Crude Extract pH 2, pH 4-1hr hydrolysate, and pH 4-12hr hydrolysate) were screened against the* Salmonella* specie with the least

MIC values were* Salmonella enterica typhimurium 2860. *Balb/C mice were infected orally with this bacterium and the extracts administered to them orally at two different doses. The efficacy of the extracts was evaluated considering bacteria load in faeces, mortality, and morbidity (body weight, physical activity, nature of the stools, signs of fever, etc.)

### 3.4. Effect of Pectin and Its Hydrolysates on Survival Rate of Salmonella-Infected Mice

**Figure 1:**
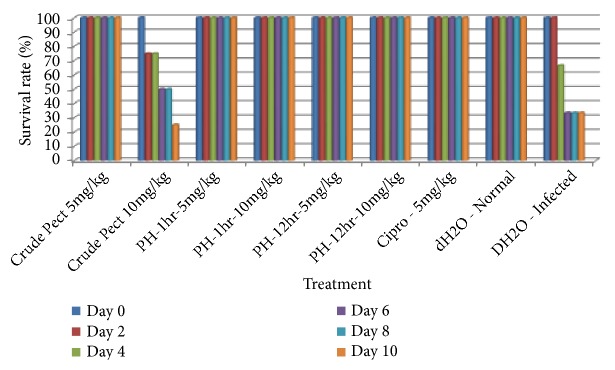
Effect of pectin and its hydrolysates on survival rate of Salmonella-infected mice.

Survival rate of animals during treatment ranged from 25% to 100% (see Figure 1). Treatment of infected mice with all extracts led to an increase in life span compared to the vehicle group, except crude pectin pH 2.5 (10mg/Kg) where 75% of the animals died.

### 3.5. Effect of the Pectin and Hydrolysates on Morbidity of Salmonella-Infected Mice

**Figure 2:**
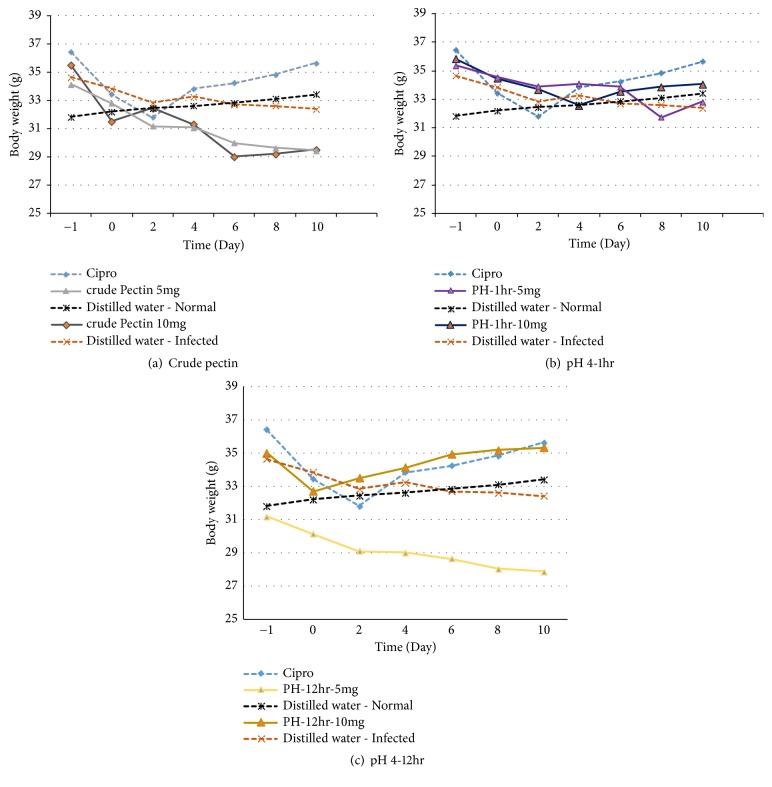
Evolution of body weight in different treatments.

*Body weight*: Figure 2 illustrate variation in body weigh with reference to the treatment. The body weight did not show any significant (*P *> 0.05) differences between the treated groups and the positive control (infected and treated with ciprofloxacin) except for crude pectin 5mg/Kg which showed significance (*P *< 0.05) from day 6 till day 10, and pectin hydrolysate 12 hours and 10mg/Kg which showed significance from day 4 till day 10.

The normal group (noninfected animals, with no treatment) witnessed a constant increase, contrary to the gradual decrease observed in infected mice receiving without treatment (receiving only distilled water). Mice who received crude pectin at 5mg/Kg lost weight up till day 2, and then a slight but steady increase till day 10. There was a drastic drop in weight for mice that were treated with crude pectin 10mg/kg. The weight loss was greater than that in the negative control. Mice treated with pH 4-1hr, 5mg/Kg experienced a decrease in body weight till day 2 and increase in day 4, a decrease in day 8, and then an increase in day 10. pH 4-1hr, 10mg/Kg treated mice had a decrease in body weight till day 4 and then a constant increase till day 10. Mice who received pH4-12hrs, 5mg/Kg experienced a decrease in body weight while those who were treated with pH 4-12hr, 10mg/Kg had a steady rise in weight and started recovering faster than animals that were treated with ciprofloxacin.

### 3.6. Effect of Pectin Extract and Hydrolysates on* S. enterica typhimurium *2860 Bacterial Load

**Figure 3:**
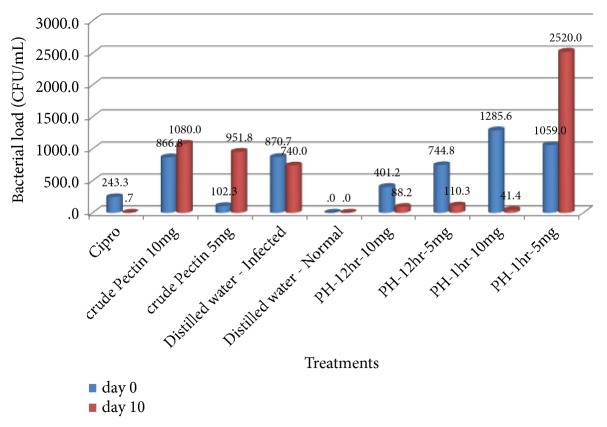
Effect of pectin extract and hydrolysates on* S. enterica* typhimurium 2860 bacterial load.

The bacteria load in mice was evaluated by colony counts per ml of* Salmonella *in 0.5g of faeces. After induction, CFUs ranged from 0 to 2720 (Figure 3). Treatment of infected mice with the extracts and ciprofloxacin significantly decreased (p < 0.05) colony counts of* Salmonella* in some groups, compared to the pretreatment values.

Figure 3 shows the change in CFU at the beginning and the end of the experiment.

During treatment three of the treated groups recorded steady decrease in bacteria load and three experienced an increase. Mice treated with crude pectin 5mg/Kg, crude pectin 10mg/Kg, and pH-1hr, 5mg/Kg experienced an increase in bacteria load while mice treated with pH-1hr, 10mg/ Kg, PH-12hr, 5mg/Kg, and PH-12hr, 10mg/Kg had a relative decrease in bacteria load.

## 4. Discussions

The emergence of multidrug resistance in* Salmonella *species has rendered most existing therapies ineffective or suboptimal for the treatment of typhoid fever. In addition to resistance induction, antibiotics are sometimes associated with opposing side effects such as hypersensitivity, immune-suppression, and allergic reactions. It is therefore important to identify new sources of antimicrobial agents for the treatment of infectious diseases such as typhoid. Pectin and pectin oligosaccharides have been identified as sources of antibacterial and antiadhesive agents especially against enteric bacteria. Pectin extracted from “ambarella” or “cas mango” and its hydrolysates were tested for their anti-*Salmonella typhi* activity* in vitro*, and the active extracts were later screened for their efficacy in infected mice. Four test organisms were used for the study, three laboratory strains and one clinical isolate.

The antimicrobial susceptibility test showed that only one out of all eleven extracts was active on all the* Salmonella *species with zones of inhibition ranging from 12 mm to 15 mm at a concentration 100*μ*g/ml. The zones of inhibition of microbial growth are a function of antimicrobial activity of the extracts. However it depends on the solubility and diffusion capacity of the tested products. Crude pectin (P^H^2.5) was the only active extract* in vitro* with a diameter of 15mm on* Salmonella* clinical and least 12mm on* S. enterica *typhimurium 2860.

From dilution method, the MIC values ranged from 5.68*μ*g/ml to 44.45*μ*g/ml. Crude pectin (P^H^2.5) had the least MIC values for all four Salmonella strains, indicating that it was the most active extract. Also, this pectin had MBC values for all four strains confirming that the extract did not only inhibit bacterial growth but could kill bacteria at certain concentrations. These results correlate with previous findings by Mathur* et al*. [5] and Niharika and Abhishek [13]. Both researchers established the antimicrobial activity of pectin extracted from apple pomace and citrus peel on* E. coli* and methicillin resistant* Staph aureus. * Pectin (pH4.0) hydrolyzed with EPG at 1 hour and 12 hours had similar MICs for all strains except* Salmonella choleraesuis* where the 1-hour hydrolysate had an MIC of 44.45*μ*g/ml and the 12-hour hydrolysate an MIC of 22.7*μ*g/ml. This suggests that both hydrolysates have almost the same potency against the* Salmonellae*. The 1-hour hydrolysate was bactericidal at 44.45 *μ*g/mL for all species except* Salmonella clinical* while the 12-hour hydrolysate was bactericidal at 22.7 *μ*g/mL for all species except* Salmonella clinical*. Thus both hydrolysates only inhibited the growth of* Salmonella clinical *but did not kill the bacteria at any concentration. Generally, it was established that the pectin oligosaccharides found in both hydrolysates had an antibacterial activity against Salmonella species in vitro by microdilution. This correlates with results obtained by Lee and Lee [16] who established that Eucosterol Oligosaccharide Isolated from Bulb of Squil Plant had antibacterial activity against* E. coli*,* Salmonella typhimurium*, and* Staphylococcus aureus*. Generally, it was observed that crude pectin (pH2.5) lost its antibacterial potency upon purification and hydrolysis while crude pectin (pH4.0) instead gained antimicrobial potency upon purification and hydrolysis. This can be explained by the fact that extraction conditions such as P^H^ and other physic-chemical processes affect the chemical characteristics of pectin from various plant tissues [2, 17–20]. Purification and hydrolysis may have destroyed or introduced some side groups on pectin and its hydrolysates which either induces potency or inhibits potency.

Based on information provided by the* in vitro *antibacterial assay and particularly the microdilution test results, an* in vivo *study was undertaken in a bid to verifying the therapeutic efficacy of the active extracts. The efficacy was evaluated considering survival rate, body weight, and bacterial load (CFUs).

Treatment of infected mice with all extracts led to an increase in life span, except crude pectin P^H^2 (10mg/Kg) where 75% of the animals died during treatment. The death of mice who received crude pectin 10mg/Kg suggests that the concentration was toxic to the mice, considering the fact that 100% of mice who received a lower dose of 5mg/Kg survived. In general, the mean body weight of all typhoid-infected groups reduced very significantly (p = 0.00) after induction of typhoid (weight range initial 30.1-40.0 (34±2.64) g, weight range after infection was 27-39g; 32.87±3.01). The decrease in body weight by all infected animals could be attributed to the various pathophysiological effects that are produced during* Salmonella* infection in mice like diarrhea, fever, and loss of appetite [20]. The body weights did not show any significant (*P *> 0.05) differences between the treated groups and the positive control (infected and treated with ciprofloxacin) except for crude pectin 5mg/Kg which showed significance (*P *< 0.05) from day 6 till day 10, and pectin hydrolyzed 12 hours which showed significance from day 4 till day 10. Except for crude pectin 10 mg/kg, all the test groups and the positive control experienced a decrease in body weight till day 2, followed by a steady recovery up to day 10. Results of the on bacteria load showed that after administration of the aqueous extracts, three of them inhibited the growth of* S. typhimurium* and thus reduced the numbers of viable bacterial CFU recovered from faeces. This reduction was dose-dependent in animals infected and treated. The marked reduction bacteria load confirmed the antimicrobial potency of the hydrolysates and therefore suggests its efficacy in the treatment of typhoid fever. These results showing the anti-Salmonella potential of pectin and hydrolysates, corroborate those obtained by Searle* et al*. [21] who established that a mixture containing galacto-oligosaccharides, produced by the enzymatic activity of* Bifidobacterium bifidum*, reduces* Salmonella enterica *serovar* typhimurium* infection in mice.

Mice treated with crude pectin 5mg/Kg, crude pectin 10mg/Kg, and pH-1hr 5mg experienced an increase in bacteria load, indicating the inefficiency of the crude pectin. Considering the above three parameters, survival rate, body weight, and bacterial clearance, the results of this study indicate pectin hydrolysate 12 hours, 10mg/Kg had the highest efficacy in the treatment of typhoid fever, followed by pectin hydrolysate 12 hours, 5mg/Kg, and then pectin hydrolysate 1 hour, 10mg/Kg.

Previous studies that involved evaluating functionalities of different oligosaccharides showed the tremendous potential of oligosaccharides in various aspects related to health and disease. Functional oligosaccharides have been found effective in gastrointestinal normal flora proliferation and pathogen suppression, dental caries prevention, enhancement of immunity, facilitation of mineral absorption, source of antioxidant, antibiotic alternative, regulators of blood glucose in diabetics, and serum lipids in hyperlipidemics [7]. Xylooligosaccharides in pharmaceutical preparations exhibit antiallergy, antimicrobial, anti-infection, and anti-inflammatory properties, selective cytotoxicity, and immunomodulatory action [10]. Haw pectin oligosaccharides (HPOS) showed dose and pH dependent antibacterial activity against* E. coli*.

Oligosaccharides have shown to also have the ability to protect against pathogen adhesion and invasion by receptor mimicry. Galacto oligosaccharide (GOS) reduced adherence of Enteropathogenic* E. coli* (EPEC) to Human HeLa contaminant carcinoma HEp-2 and the heterogeneous human epithelial colorectal adenocarcinoma Caco-2 cells* in vitro*; the antiadhesive activity of GOS was reported to be more effective than of both FOS and inulin [22]. Similarly, GOS was found to reduce the invasion of* S. Typhimurium* SL1344 and LT2 to HT29 cells lines [21]. Furthermore, pectin and pectic oligosaccharides reduced the activity of* E. coli* O157:H7 produced shiga toxin, likely by inhibiting binding of the toxin [10]. From the present study, two pectin hydrolysates were identified from “cas mango” (*Spondias dulcis*), namely, the p^H^4 1 hour and 12 hours hydrolysates, with significant activity and bactericidal effect on all four* Salmonella *species tested* in vitro,* and confirmed therapeutic effects on* Salmonella typhimurium* Balb/C mouse model of typhoid.

## 5. Conclusions

These findings are therefore a clear indication that pectin oligosaccharides might be a valuable and yet explored source of new anti-Salmonella therapies. Further investigations are required to fully characterize these oligosaccharides and gather more inside on their mechanism of action, as well as their safety.

## Figures and Tables

**Table 1:** Description of the different experimental groups.

Group	Number	Treatment
A: Test 1	5	Crude pectin pH2.5,5mg/Kg
B: Test 2	5	Crude pectin pH2.5, 10mg/Kg
C: Test 3	5	Hydrolyzed pectin pH 4, 1h, 5mg/Kg
D: Test 4	5	Hydrolyzed pectin pH 4, 1h, 10mg/Kg
E: Test 5	5	Hydrolyzed pectin pH 4, 12h, 5mg/Kg
F: Test 6	5	Hydrolyzed pectin pH 4, 12h, 10 mg/Kg
G: Neutral	5	Distilled water
H: Positive control	5	Ciprofloxacin (mg/Kg)
I: Negative water	5	Distilled water

**Table 2:** Physicochemical characteristics of the different pectin extracts and their hydrolysates.

Pectin	Procedure	Pectin solution	Pectin hydrolysate
P^H^ 2.5	15 minutes after addition of KOH	Formation of transparent semi-gel	No formation of transparent semi-gel
	Acidification with dilute HCl	Gelatinous precipitate is formed	Gelatinous precipitate is not formed
	Boil precipitate for 5 minutes at 100°C	Precipitate becomes cloudy	Precipitate does not become cloudy

P^H^ 4	15 minutes after addition of KOH	Formation of transparent semi-gel	No formation of transparent semi-gel
	Acidification with dilute HCl	Gelatinous precipitate is formed	Gelatinous precipitate not formed
	Boil precipitate for 5 minutes at 100°C	Precipitate becomes cloudy	Precipitate does become cloudy

Commercial pectin	15 minutes after addition of KOH	Formation of transparent semi-gel	No formation of transparent semi-gel
	Acidification with dilute HCl	Gelatinous precipitate is formed	Gelatinous precipitate is not formed
	Boil precipitate for 5 minutes at 100°C	Precipitate becomes cloudy	Precipitate does not become cloudy

**Table 3:** *In vitro* inhibition effect of pectin extract/hydrolysate on the growth of different Salmonella strains.

Extract / Hydrolysate	*Salmonella enterica*	*Salmonella enterica*	*Salmonella *	*Salmonella *
	*typhimurium *2860	*typhimurium *2488	*spp.* clinical	*choleraesuis*
	(mm)	(mm)	(mm)	(mm)
Commercial pectin	6	6	6	6
Crude pectin P^H^ 4	6	6	6	6
Crude pectin P^H^ 2.5	12	13	15	13
Pure pectin P^H^ 2.5	6	6	6	6
Pure pectin P^H^4	6	6	6	6
Hydrolyzed commercial Pectin 1hr	6	6	6	6
Hydrolyzed P^H^4-1hr	6	6	6	6
Hydrolyzed P^H^4-12hr.	6	6	6	6
Hydrolyzed P^H^2.5-1hr	6	6	6	6
Hydrolyzed P^H^2.5-12hr.	6	6	6	6
Hydrolyzed commercial-12hr.	6	6	6	6
Negative control	6	6	6	6
Ciprofloxacin (10*µ*l/ml)	31	30	22	34
Ciprofloxacin (50*µ*l/ml)	40	41	28	43

**Table 4:** MIC and MBC values for the extracts on *Salmonellae*.

Extract/Hydrolysate	*S. enteric*		*S. enteric*		*Salmonella spp.*		*S. choleraesuis*	
	*typhimurium* 2860		*typhimurium* 2488		clinical isolate			
	MIC	MBC	MIC	MBC	MIC	MBC	MIC	MBC
	(*μ*g/ml)	(*μ*g/ml)	(*μ*g/ml)	(*μ*g/ml)	(*μ*g/ml)	(*μ*g/ml)	(*μ*g/ml)	(*μ*g/ml)

Commercial pectin	>1	ND	>1	ND	>1	ND	>1	ND
Crude Extract P^H^ 4	>1	ND	>1	ND	>1	ND	>1	ND
Crude Extract P^H^2	11.36	11.36	5.68	11.36	11.36	22.7	5.68	11.36
Pure Pectin P^H^ 2	>1	ND	>1	ND	>1	ND	>1	ND
Pure Pectin P^H^ 4	>1	ND	>1	ND	>1	ND	>1	ND
Hydrolyzed commercial Pectin 1hr	>1	ND	>1	ND	>1	ND	>1	ND
P^H^ 4 hydro 1hr	22.7	44.45	22.7	44.45	22.7	No MBC	44.45	44.45
P^H^ 4 hydro 12hr	22.7	44.45	22.7	No MBC	22.7	No MBC	22.7	44.45
P^H^ 2 hydro 1hr	>1	ND	>1	ND	>1	ND	>1	ND
P^H^ 2 hyd 12hr.	>1	ND	>1	ND	>1	ND	>1	ND
Negative control	>1	ND	>1	ND	>1	ND	>1	ND
Positive control	<11	<11	<11	<11	<11	<11	<11	<11

ND = not determined.

## Data Availability

The datasets used and/or analyzed during the current study are available from the corresponding author upon reasonable request.

## Abbreviations

CFU: Colony forming unit
GOS: Galactooligosaccharide
HPOS: Haw pectin oligosaccharides
MBC: Minimum bactericidal concentration
MIC: Minimum inhibitory concentration
UB-IACUC: University of Buea Ethical Review Board for the Use of Animals in Research.
